# Cough

**Published:** 1884-03

**Authors:** 


*Cough* is not an early symptom of consumption, necessarily, for there are many cases on record, in which cough was not an observed symptom, until within two or three weeks of death, and on examination, the lungs presented a diseased mass, burrowed with cavities.

*Spitting blood* is not an early symptom of consumption, necessarily, for about one-third of those who die of that disease, do not spit blood at all.

Among the very earliest symptoms of forming consumption, are combinations of the following; not all, perhaps, observable in any one case: A quicker pulse than common, a paler face, easily chilled after eating, more readily put out of breath than common, less fullness of flesh than usual at the corresponding season of the year, an unusual feeling of unrest on getting up in the morning, a greater tendency to coldness of the hands and feet, every now and then a day passing without any action of the bowels, with a very bad taste in the mouth when first waking up in the morning; a cold is easily taken, is more frequent, and lasts longer and longer, until one cold runs into another, making the confirmed cough, so ominous of approaching ill.

It will be seen at a single glance, from these symptoms, that they all indicate one thing, and that one thing is at the bottom of every case of consumption—*a want of vitality*; that is, a want of general vigor of system, of constitution.

But of all the things named, it will be more practical to select the two which are seldom, if ever absent, in any of the above combinations which result in consumption; hence it is important to be at some pains in stating them in their bearings. A quick pulse and a short breath pervade the disease from its earliest beginnings, during its entire progress, and down to its fatal end. Multitudes of lives might be saved yearly, if these two symptoms were promptly and wisely attended to. The importance of so doing, no language can adequately portray, and if it did, the people would not attend to it, with only here and there an exception. But a great truth is of small seed and of slow growth; yet that growth is certain, and its spread uncontrollable—the more so, as education becomes more general.

